# Glycomic Expression in Esophageal Disease

**DOI:** 10.3390/metabo2041004

**Published:** 2012-11-21

**Authors:** Sanjay Mohanty, Athanasios Tsiouris, Zane Hammoud

**Affiliations:** Henry Ford Hospital, 2799 W. Grand Blvd., Detroit, MI 48202, USA; Email: smohant1@hfhs.org (S.M.); atsiour1@hfhs.org (A.T.)

**Keywords:** esophageal adenocarcinoma, glycomics, glycoproteomics, biomarkers, cancer

## Abstract

Glycosylation is among the most common post translation modifications of proteins in humans. Decades of research have demonstrated that aberrant glycosylation can lead to malignant degeneration. Glycoproteomic studies in the past several years have identified techniques that can successfully characterize a glycan or glycan profile associated with a high-grade dysplastic or malignant state. This review summarizes the current glycomic and glycoproteomic literature with specific reference to esophageal cancer. Esophageal adenocarcinoma represents a highly morbid and mortal cancer with a defined progression from metaplasia (Barrett's esophagus) to dysplasia to neoplasia. This disease is highlighted because (1) differences in glycan profiles between the stages of disease progression have been described in the glycoproteomic literature; (2) a glycan biomarker that identifies a given stage may be used as a predictor of disease progression and thus may have significant influence over clinical management; and (3) the differences in glycan profiles between disease and disease-free states in esophageal cancer are more dramatic than in other cancers.

## 1. Introduction

It is estimated that up to fifty percent of proteins in humans undergo post-translational modifications (PTMs) [1]. These modifications are essential to the function and character of certain kinds of proteins. It has been known for decades that specific alterations in PTMs can lead to pathology including tumor invasion and metastasis [2,3]. Indeed, several glycoproteins are already used clinically as indicators of the presence of disease or as disease surveillance—famously, these include prostate specific antigen (PSA), Her2/Neu, CA 125, and CEA [4].

Esophageal adenocarcinoma is a cancer that has been rising in incidence faster than any other cancer in the United States [5]. It is a disease that follows a well-defined progression from metaplasia to dysplasia to neoplasia. Surgical resection in appropriately selected patients remains the mainstay of treatment. Mortality continues to be high, due in part to the typically advanced nature of the disease at the time of diagnosis. At present, the diagnosis of esophageal cancer is established on the basis of an endoscopic biopsy with histologic examination. A biomarker—in particular, a biomarker that could distinguish a patient with high-grade dysplasia from one with frank neoplasia—would have significant influence over the subsequent clinical management and decision-making process following a diagnosis.

The fields of glycomics and glycoproteomics, both closely aligned with the broader field of proteomics, seek to identify and characterize these known and aberrant changes in glycosylation on a large scale using novel isolation and identification techniques that can be variably combined and sequentially organized depending upon the specific question [6]. From the standpoint of cancer, aberrantly glycosylated proteins present an interesting opportunity for biomarker identification. Advances in the technology used to study these molecules have been made over the last decade, which have allowed the delineation of specific alterations in glycosylation that may, in the future, be exploited in order to identify malignant degeneration in a clinical setting. The complexities and challenges of glycomics remain considerable due to technologic obstacles and the peculiarities of the molecules themselves—the presence of distinct iterations of glycosylation for a given glycoprotein and their relative concentration in biologic fluids, for example. The significance of glycans in cancer stems in part from their role as extracellular receptors and participants in normal cell-to-cell interaction, which are presumably deranged in cancer. 

Glycosylation changes in cancer have been shown to have a variety of different forms. In general, most studies indicate changes in the number or structure of saccharide moieties. Alterations, and increases in particular, in both sialyation and fucosylation have been described, as have increases in the number of branched oligosaccharides. Within the last several years, a number of authors have described techniques using novel technologies, such as mass spectroscopy, that have shown promise in using these known alterations in glycosylation to isolate both specific glycoprotein profiles and individual glycans in breast, colorectal, hepatocellular, pancreatic, prostate, ovarian, and esophageal cancer [4,7,8,9,10,11,12].

In this review, we highlight glycoproteomics as it relates to esophageal adenocarcinoma. Glycoproteomic profiles of patients with esophageal diseases along the metaplasia-neoplasia continuum have not only shown distinction from other disease models, but certain techniques have demonstrated that these profiles may be used in the future to distinguish a particular time point along the metaplastic-neoplastic continuum. This has broad and significant implications for management decisions and presumably for outcomes.

## 2. Scientific Principles

Proteomic studies, or the large-scale study of an organism’s serum protein profile, have shown that these profiles can differ in real and detectable ways between diseased and disease-free states. The first studies showing potential for biomarker discovery demonstrated measurable differences in protein profiles between disease free and disease states, specifically with regard to prostate and ovarian cancers [13,14]. Diamandis et al [15,16] noted problems with these studies and others like it—namely, that the process of identifying proteins of interest was notoriously sensitive to variations in sample preparation and storage and various other factors such as gender, age, and co-morbid conditions. Subsequent validation studies of these initial proteomic studies were shown to have introduced bias. Additionally, the complexity of protein production and regulation make it very challenging to identify and characterize the specific constituents of different protein peaks, leading to interest in more granular studies of so-called subproteomes, exemplified by post-translational modifications and specifically, glycosylated proteins.In humans, many proteins undergo one or more types of post-translational modifications (PTMs) such asubiquination, phosphorylation, acetylation, and glycosylation. Some authors have noted the inadequacy and inaccuracy of grouping all glycosylation together, highlighting the diversity and number of types of glycosylation not shared by other post-translational modifications [17]. Glycoproteomics focuses on the examination of proteins that have undergone glycosylation. These modifications play an important role in the study of disease, for they are among the most numerous and most structurally diverse post-translational modifications. Their incredibly small concentrations in biologic systems as well as this structural diversity make them challenging to study. They also offer several levels upon which investigators may choose to focus and characterize a given glycoprotein or glycan of interest.

Protein glycosylation is organized broadly into two categories based upon the amino acid residue to which the glycan is attached. N-glycans are attached to an asparagine, O-glycans are attached to OH groups, generally on either a serine or threonine residue. There are a number of ways that these structures can be studied, and in fact, much of the work of the last several years has focused on the specifics of determining which technique to use at a particular point of the workflow. At each level of a study, there are a number of expanding and ever-evolving and improving techniques which makes the field particularly dynamic. These structures can be studied as intact glycopeptides or after cleavage from the protein as glycans, at which point investigators can endeavor to identify, structurally characterize and profile them. The challenges of studying intact glycoproteins are considerable and have been taken on by the field of glycoproteomics. Because this way of studying these important structures encompasses information about peptide-glycan binding sites, much effort has been spent on improving upon and modifying proteomic techniques in order to apply them to glycopeptides. Enzymatically releasing glycans from glycopeptides is more commonly done, but ideally in the future, the ability to do both in conjunction would provide a wealth of information and a more complete picture of how exactly structure impacts function in these structures. 

The challenges of glycomics and glycoprotemics remain related to the inherent complexity of biologic systems. Much of the difficulty with broader studies of an organism’s proteome is the difficulty that comes with attempts to detect molecules that are small in amount or concentration but may be essential to function. In broad strokes, the workflow for isolating and then characterizing glycans has been well-established. Summaries of the varieties and relative strengths and weaknesses of techniques at a given stage of glycan characterizations are beyond the scope of this article and can be found elsewhere [6]. We will briefly review those used in cancer biomarker glycomic studies and then focus on esophageal adenocarcinoma. 

As many biologic samples of interest (serum, plasma, tissue, *etc.*) are incredibly complex combinations of proteins and other molecules, separation and removal of abundant and otherwise nonspecific and “noisy” components steps is generally necessary to start. Next, glycoproteins of interest are isolated using a number of different techniques. Though glycans can be studied as part of an intact glycopeptide, most of the studies with regard to cancer have studied N-glycans after they have been enzymatically released using amidases. Other authors have remarked on the shortcomings of analysis techniques which focus on released glycans, as glycan-peptide binding site information is lost [6]. The glycan-peptide linkage on which glycoproteomics is focused has been exploited to tell an interesting story about patients with a spectrum of esophogeal diseases. However, the study of intact glycoproteins is difficult due to more numerous and complex interactions between components of the peptide portion of the molecule with the mass spectrometer, impairing its ability to accurately characterize the glycan portion.

Most conventional fractionation techniques can be used in the initial stages of the typical glycomic or glycoproteomic workflow to remove highly abundant proteins, non-glycosylated proteins and other “noisy” molecules. It is in the stages that follow that several techniques have been pioneered on the basis both of chemical and lectin/antibody affinity principles for more specific identification and isolation. This is typically done by way of enrichment of specific types of glycosylated proteins. Hydrazide coupling exploits the presence of *cis* diols in monosaccharides. After a chemical modification, such as oxidation to an aldehyde, the glycan is coupled to hydrazide groups on static beads. In this way, glycans of interest are immobilized, which enables the completion of subsequent washing stages to eliminate non-bound molecules. Agarose enrichment, another technique which exploits basic biochemical principles, isolates glycoproteins by immersing them in a hydrophobic environment with agarose gel. The environment emphasizes hydrophilic interactions between glycoproteins of interest and the agarose media, allowing unbound molecules to be washed. Lectin affinity techniques and related antibody-based techniques represent perhaps the most exciting development in glycomics in the last decade. Lectins are proteins isolated from various organisms including plants, fungi, and animals, which exhibit special affinity for carbohydrates. By targeting specific glycans that are differentially expressed in diseased states when compared to the disease-free state, this technique has provided much excitement with regard to identification of potential cancer biomarkers.

## 3. Esophageal Adenocarcinoma

Esophageal adenocarcinoma (EAC) remains a highly morbid and mortal malignancy despite recent advances in our understanding of its molecular biology; minimally invasive surgical techniques; surveillance and screening practices; and chemotherapeutic options. 

Adenocarcinoma in particular has been increasing in incidence and now accounts for over fifty percent of esophageal cancer in Western countries. It is a cancer that has a well-established and characteristic progression from metaplasia to dysplasia to neoplasia. Barret’s esophagus, the eponym given to esophageal mucosa which has undergone a metaplastic change to a columnar histology, occurs as complication in up to fifteen percent of patients with gastroesophogeal reflux disease. Efforts to find a biomarker that mimics this process in order to better predict those patients who would derive the most survival benefit from surgery have been ongoing. Endoscopic surveillance is typically used in patients with known Barrett’s esophagus; a tissue diagnosis of adenocarcinoma is typically obtained via endoscopic methods. Unfortunately, a tissue diagnosis of adenocarcinoma is typically obtained late in the course of the disease, and the work-up at that point is focused on identifying evidence of local invasion and/or metastatic disease. Metastatic disease and advanced local disease preclude a surgical option. Thus, finding a biomarker that could potentially identify a metaplastic or dysplastic process would then ideally be able to identify patients who would derive the most benefit from surgical resection.

## 4. Esophageal Disease and Glycomics

In two glycan-based studies, Hammoud *et al*. and Mechref *et al*. [18,19] demonstrated the first comparative glycomic profile in a model of esophageal cancer. In their study, glycans isolated from whole blood samples of patients with documented Barrett’s esophagus, Barrett’s esophagus with high grade dysplasia, esophageal adenocarcinoma, and patients without disease—a sample pool mimicking the spectrum from metaplastic to neoplastic disease in the esophagus—were enzymatically released using PNGase F. Following this, solid phase extraction and permethylation labeling of purified N-glycans of interest was conducted. The final characterization step used matrix-assisted laser desorption ionization/time of flight mass spectroscopy. With the help of bioinformatic analysis, eight glycans were found to be characteristically distinct in three separate comparisons: esophageal adenocarcinoma and normal; esophageal adenocarcinoma and Barrett’s esophagus plus high grade dysplasia; and control and Barrett’s esophagus plus high grade dysplasia. 

Several interesting insights can be taken from this study. First, the glycan profiles between the four sample groups were found to be distinct. The distinction between esophageal adenocarcinoma and the normal control was notable; but so too was the distinction between known precursor lesions and high grade dysplasia. Admittedly, this difference was much less than that observed between the neoplastic and metaplastic lesions. Nevertheless, the limited difference exhibited here would suggest that there is a possibility that some detectable differential glycan or more sensitive glycoprotein profile could be defined as a biomarker with significant influence over clinical management. Second, the particular difference between the adenocarcinoma and normal samples was a decrease in total fucosylation of N-glycans, this was also noted between the two Barrett’s esophagus samples (with and without high-grade dysplasia), though this did not reach statistical significance. This decrease in fucosylation seems to be unique to esophageal adenocarcinoma—other glycoprotein studies with applications to cancer have described increases in fucosylation as well as sialyation aberration—and the authors thus postulated that glycosylation changes may be cancer specific. Fucosylation as a specific type of glycosylation has been known to play an important role in cancer, with studies finding that increases in fucosylation are associated with hepatocellular and pancreatic, cancer and that perhaps represent a fruitful area of research within glycomics for the discovery of cancer biomarkers and new cancer therapies [20]. Finally, the group was able to continue the work done in this exploratory study in a second study, which sought to further quantify the fucosylation changes described.

In the subsequent glycoprotein-based study, Mann *et al.* [21] obtained samples from three sample pools. Two of the sample pools were representative of esophageal disease states along the spectrum of dysplastic-neoplastic processes discussed above. One sample was taken from patients with high grade dysplasia; a second from patients with esophageal adenocarcinoma; and a third comparison sample taken from patients without any disease. Using immunoaffinity chromatography the authors removed highly abundant proteins. After this, serial lectin affinity chromatography was used with two separate lectins—*aleuria aurantia lectin* and *lotus tetrgonolobus lectin*—in a sequential fashion to enrich glycoproteins. These two lectins are fucose-specific. Following treatment with the former, the unbound fraction was removed and treated with the latter. The final steps included treatment with trifluoroacetic acid and typsin digestion of the isolated glycoproteins before characterization was carried out with liquid chromatograph-mass spectroscopy.

This study yielded important results that distinguish each of the three comparison groups with one another. A 1.5–2 fold difference was measured between each of the groups. Of particular interest was in the high-grade dysplasia (HGD) and adenocarcinoma groups. Each showed the same series of six proteins that were upregulated when compared to the normal, leading to the conclusion that progressive increases in the amounts of the identified proteins could be indicators of disease progression. In addition, the authors identified and highlighted three glycoproteins, two of which showed increases in both HGD and EAC. These included Fetuin-B, a cystatin with unknown function; EMILIN-2, a matrix glycoprotein and possible component of the host immune response to cell dysplasia; and Collagen alpha-1(I) chain, another matrix and structural protein, the overexpression of which has been noted in breast, colon, prostate, and skin cancers. The technique used by the study authors, which combined removal of highly abundant proteins with some the latest and continually evolving protein fractionating and mass spectroscopic techniques yielded specific protein identification and put forward the possibility that similar techniques could be exploited to examine other models of cancer and of disease progression.

## 5. Conclusions

Glycomics and glycoproteomics represent two very exciting fields that have the potential to impact the way clinicians screen for and manage certain kinds of cancers in the next few decades. Much work remains**,** and the challenges of the next few years will be largely those related to refining both the sensitivity of individual separation and identification techniques and the way in which certain kinds of techniques can be combined in a sequential or simultaneous fashion in order to give a desired level of granularity. 

The enrichment methods in models of esophageal disease are an illustration of the potential of glycomic-based studies and their role in the identification of biomarkers and biomarker profiles in patients with precancerous and cancerous conditions [22,23,24,25,26]. Studies within the last year continue to demonstrate on a more nuanced level the nature of the differences in glycosylation**,** particularly in breast and ovarian cancer [27,28]. As these studies continue and those changes in glycosylation unique to cancer in general and to specific types of cancer in particular are further characterized**,** the identification of biomarkers will follow.
